# Ginsenosides induce extensive changes in gene expression and inhibit oxidative stress-induced apoptosis in human lens epithelial cells

**DOI:** 10.1186/s12906-020-2826-8

**Published:** 2020-02-11

**Authors:** Zhewen Wang, Shiping Zhou, Xiaoqing Hu, Jiannan Chai

**Affiliations:** 1 Department of Ophthalmology, First Hospital of Jilin University, No.1 Xinmin Avenue, Changchun, 130021 China; 2 Endoscopy Center, First Hospital of Jilin University, Changchun, 130021 China; 3 Department of Clinical Laboratory, First Hospital of Jilin University, No.1 Xinmin Avenue, Changchun, 130021 China

**Keywords:** Ginsenosides, HLE-B3 cells, H_2_O_2_

## Abstract

**Background:**

The effect of ginsenosides on the growth and apoptosis of human lens epithelial (HLE) B3 cells exposed to H_2_O_2_ was investigated. In addition, the effect of ginsenosides on gene expression in HLE-B3 cells was analyzed using microarray assays to determine its molecular mechanism.

**Methods:**

HLE-B3 cells were treated with 1.75 M H_2_O_2_ in the presence or absence of 5, 10 or 20 μM ginsenosides. Cell viability and apoptosis were examined by MTT assays and flow cytometry, respectively, at 24 to 120 h after the treatment. Furthermore, HLE-B3 cells were treated with 20 μM ginsenosides for 8 days and total RNA was isolated and analyzed using the Affymetrix GeneChip Array. Principal component analysis was performed to visualize the microarray data.

**Results:**

Addition of ginsenosides significantly alleviated the growth inhibitory effect of H_2_O_2_ on HLE-B3 cells and the percentage of viable cells was increased by more than 3 folds. Flow cytometric analysis showed that 6.16 ± 0.29% of H_2_O_2_-treated HLE-B3 cells were early apoptotic cells, and the percentage was reduced to 4.78 ± 0.16% (*P* < 0.05) in the presence of 20 μM ginsenosides. Principal component analysis revealed that ginsenoside caused extensive changes in gene expression in HLE-B3 cells. A total of 6219 genes showed significant differential expression in HLE-B3 cells treated with ginsenoside; among them, 2552 (41.0%) genes were significantly upregulated, whereas 3667 (59.0%) genes were significantly downregulated. *FOXN2*, *APP* and *RAD23B* were the top three upregulated genes while *WSB1*, *PSME4* and *DCAF7* were the top three downregulated genes in HLE-B3 cells treated with ginsenosides.

**Conclusion:**

Ginsenosides induce extensive changes in the expression of genes involved in multiple signaling pathways, including apoptotic signaling pathway and DNA damage response signaling pathway. Ginsenosides alleviate H_2_O_2_-induced suppression of the growth of HLB cells and inhibit H_2_O_2_-induced apoptosis of HLB cells.

## Background

It has been estimated that 95 million people worldwide are affected by cataracts [1]. Age-related cataracts remain highly prevalent in the elderly population, and elderly patients with cataracts account for a significant proportion of visually impaired elderly people globally [2]. Surgery has been shown to be effective for cataract correction, but is not without risks and problems [3]. Currently, there is a lack of effective alternative treatment modalities for cataract surgery. Although many risk factors for cataractogenesis have been identified, such as long-term corticosteroid use [4], smoking, excessive UV-B exposure, and diabetes [5], other than a healthy lifestyle such as smoking cessation, there are no effective preventive measures, including pharmacological treatment of cataract formation.

Cataract is a multifactorial eye disease. Although the exact molecular mechanism of cataractogenesis remains elusive, oxidative stress (OS) has been implicated as the main culprit of cataract lens opacity [6]. Hydrogen peroxide (H_2_O_2_), a non-free radical member of the active oxygen family, is the major intracellular reactive oxygen species (ROS) in the aqueous humor, which generates hydroxyl radicals that irreversibly damage the lens epithelium. It can activate multiple signaling events such as the activation of apoptosis-associated molecules or pathways, including caspases, the Bcl-2 family, the mitogen-activated protein kinases (MAPKs), and NF-*к*B pathways, which lead to apoptosis of lens epithelial cells (HLE), ultimately resulting in lens opacification and subsequent cataract development [7, 8]. A variety of antioxidant nutrients, such as flavonoids, phenolic acids, carotenoids, and vitamins, have been tested for their ability to prevent or delay cataract development in animal studies, but their protective effects have not been demonstrated unequivocally [9].

Ginsenosides, also known as ginseng saponins, are isolated from the total saponins of *Panax notoginseng* and have been tested against various diseases including ischemic stroke [10]. Ginsenosides have antioxidant and antioxidant-related properties in a variety of cell types. Ginsenosides were shown to significantly inhibited UV-B-induced ROS elevation in HaCaT keratinocytes [11]. Ginsenoside Rg1 mediated by ultrasound-targeted microbubble destruction can reduce the level of OS, relieve intraocular pressure and reduce ganglion cell damage in glaucomatous optic nerve of rabbits. Ginsenosides Rb1 and Rd. were also shown to protect the retina from intense light-induced degeneration in BALB/c mice exposed to intense light [12]. However, the possible effect of ginsenosides on cataracts has not been examined. Given that ginsenosides have been shown to protect against UV-B exposure in keratinocytes [13, 14], we hypothesized that ginsenosides may also exert protective effects against OS-induced lens epithelial damage. In the current study, we investigated the effect of ginsenosides on the growth and apoptosis of human lens epithelial (HLE-B3) cells exposed to H_2_O_2_. To further determine the molecular mechanism of ginsenosides, we analyzed the effects of ginsenosides on gene expression in HLE-B3 cells using microarray analysis.

## Methods

### H_2_O_2_ treatment

Human lens epithelial (HLE-B3) cells purchased from American Type Culture Collection were cultured in minimal essential medium (MEM) containing 20% of fetal bovine serum in a humidified incubator at 37 °C with 5% CO_2_. Cells at 50–60% confluency were treated with H_2_O_2_ at concentrations from 0.039 to 2.5 μM for 24 h, and the concentration was incremented by a factor of two. Cell viability was examined by the 3-(4,5-dimethyl-2-thiazolyl)-2,5-diphenyl-2-H-tetrazolium bromide (MTT) assays using Cell Proliferation Reagent Kit I (MTT; Roche Applied Science) following the manufacturer’s protocol and as previously described [15]. Each experiment was repeated at least three times independently in quintuplicate. Optical density (OD) was measured at 490 nm using a microplate reader (iMark, USA). A standard curve was drawn to determine IC_50_ of H_2_O_2_. All cells were treated with H_2_O_2_ at IC_50_ for 24 h in all subsequent experiments.

### Ginsenoside treatment

HLE-B3 cells (passage 6) were plated at 6 × 10^3^ cells per well in 96-well plates. After the medium was changed, the cells at 30–40% confluency were treated with H_2_O_2_ at IC_50_ in the absence or presence of 5, 10 or 20 μM ginsenoside (Fleton Natural Products, Chengdu, China; HPLC grade pure 99.8%). Ten microliter of MTT (at a final concentration of 5 mg/ml) was added to each well and the cells were incubated for another 4 h, and the supernatant was discarded. DMSO (150 μL) was added to each well to dissolve the precipitate. Cell viability was examined by MTT assays after 24 to 120 h of drug treatment following the manufacturer’s instructions (Sigma, St. Louis, MO, USA). Each experiment was repeated at least three times independently in quintuplicate.

### Flow cytometry

Flow cytometry was performed as previously described [16]. Briefly, HLE-B3 cells were seeded in 6-well plates. When the cells were 70% confluent (5 × 10^5^ cells), they were treated with 5, 10 or 20 μM ginsenoside for 48 h and harvested. The treated cells were washed once with phosphate-buffered saline (PBS), trypsinized, and washed again in PBS containing 2% fetal bovine serum and fixed in ice-cold ethanol for at least 1 h at − 20 °C. The cells were washed, and stained with FITC-annexin V (Beyotime Biotechnology Research Institute, China) and propidium iodide (30 μg/mL) and treated with RNase (0.6 mg/mL) in PBS containing 0.5% (v/v) Tween 20 and 2% fetal bovine serum. Fluorescence-activated cell sorting analysis was performed on a FACS Calibur flow cytometer (BD Biosciences) using Cellquest software, and the Mod-Fit program (Verity Software House Inc., Topsham, ME) was used to analyze the percentage of apoptotic cells.

### Microarray

HLE-B3 cells were treated with 20 μM ginsenoside for 8 days. Total RNA was extracted using the Recover All™ Total Nucleic Acid Isolation Kit (Ambion, AM1975) following the manufacturer’s protocol. RNA was biotin-labeled using the FlashTag™ Biotin HSR RNA Labeling Kit (Affymetrix). An input of 400 nanograms of total RNA was used for each reaction. Hybridization, washing and staining were performed using the commercially available Affymetrix GeneChip Hybridization, Wash and Stain Kit. All samples were hybridized to the Affymetrix GeneChip Array. Expression data were normalized using the robust multi-array average (just RMA) method where the raw intensity values were background-corrected, log_2_-transformed and then quartile-normalized. A linear model was fitted to the normalized data to obtain a measure of expression for each probe set on each array.

### Gene analysis

Principal component analysis was carried out to visualize the microarray data. All genes were plotted on the first and second principal components. The first principal component (PC1) measured the grand mean expression and the second (PC2) measured the ginsenosides-induced expression changes. In addition, a scatter plot of principal component analysis of differential gene expression patterns in ginsenosides-treated cells and control cells was drawn. The Pearson correlation of gene expression patterns in HLE-B3 cells treated with ginsenoside was calculated between the vectors pointing from the overall mean of the entire dataset and the respective group mean. For hierarchical clustering, genes in the dataset were subjected to complete-linkage hierarchical clustering using a Euclidean distance metric. The pathway and function analyses were performed using KEGG and Gene Ontology (GO).

## Results

### Ginsenoside reverses H_2_O_2_-induced growth inhibition of HLE-B3 cells

**Fig. 1:**
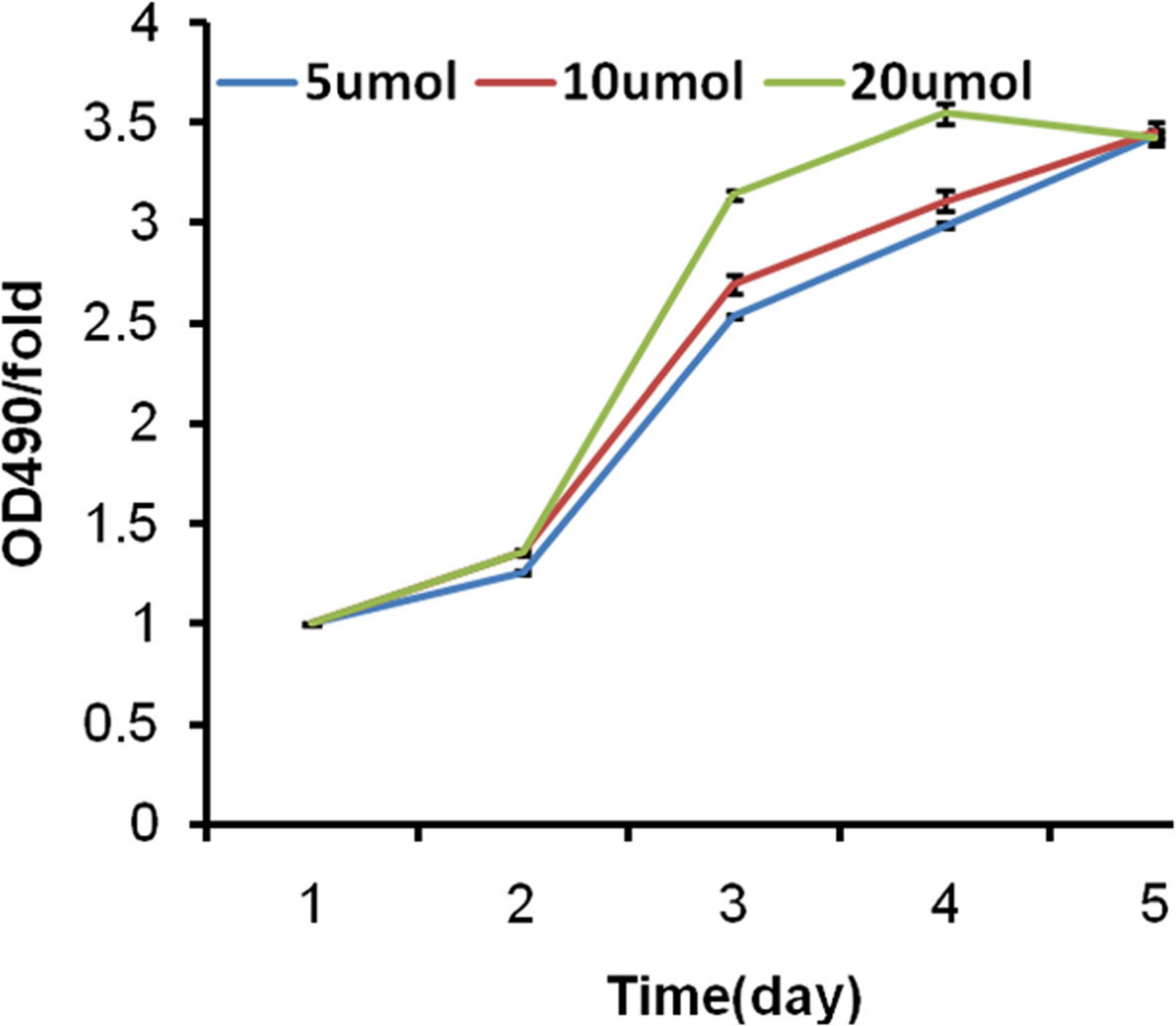
Ginsenoside reverses H_2_O_2_-induced growth inhibition of HLE-B3 cells. HLE-B3 cells were treated with 1.75 μM H_2_O_2_ and low (5 μM), mid (10 μM) and high dose (20 μM) of ginsenoside. Viabilities of HLE-B3 cells were examined by MTT assays as detailed in Methods. Ginsenoside significantly alleviated the growth inhibitory effect of H_2_O_2_ on HLE-B3 cells with more than 3-fold increase in the percentage of viable HLE-B3 cells. No statistical difference was observed in the percentage of viable HLE-B3 cells treated with low (5 μM), mid (10 μM) and high dose (20 μM) of ginsenoside (*P* > 0.05)

MTT assays showed that H_2_O_2_ exhibited a dose-dependent inhibitory effect on the viability of HLE-B3 cells. The mean inhibition rate of H_2_O_2_ steadily increased from 6.6% at a concentration of 0.039 μM to 15.3% at a concentration of 1.25 μM and rapidly reached to 85.4% at the final concentration of 2.5 μM (Fig. 1). The IC_50_ of H_2_O_2_ was 1.75 μM (ranging from 1.59 to 1.927 μM). Addition of ginsenosides significantly alleviated the growth inhibitory effect of H_2_O_2_ (1.75 μM) on HLE-B3 cells and the percentage of viable cells was increased by more than three folds (Fig. 1). There was no statistical difference in the percentage of viable HLE-B3 cells treated with low (5 μM), mid (10 μM) and high (20 μM) dose of ginsenosides (*P* > 0.05).

### Ginsenosides induce extensive changes in gene expression

**Fig. 2:**
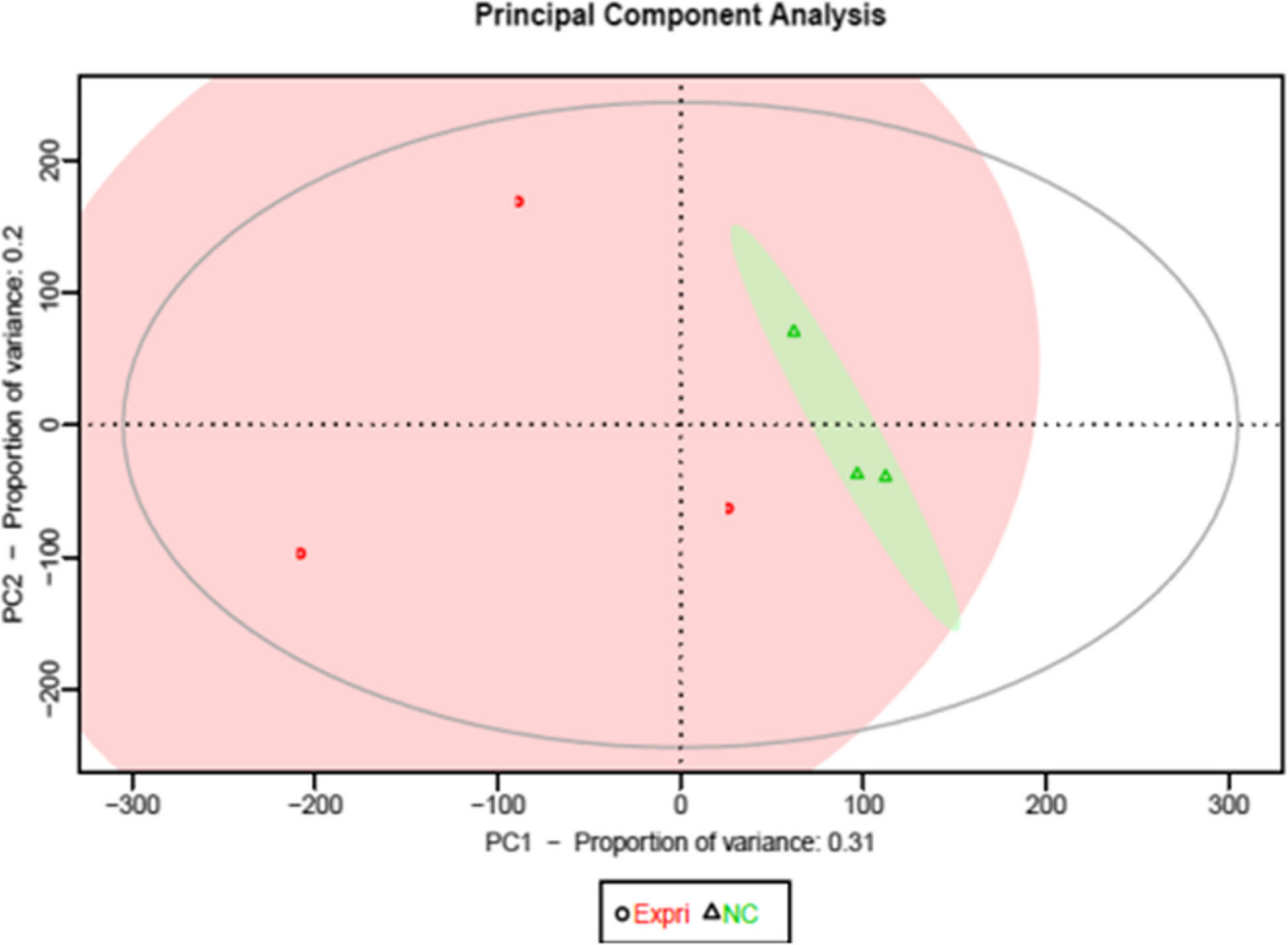
Principal component analysis for visualization of microarray data. All genes are plotted on to the first and second principal components. The first principal component (PC1) measures total average expression, and the second (PC2) measures changes in expression induced by ginsenoside. The ellipse at the center contains 95% of the genes. Pink color represents genes from ginsenoside-treated cells and green color indicates genes from control cells

**Fig. 3:**
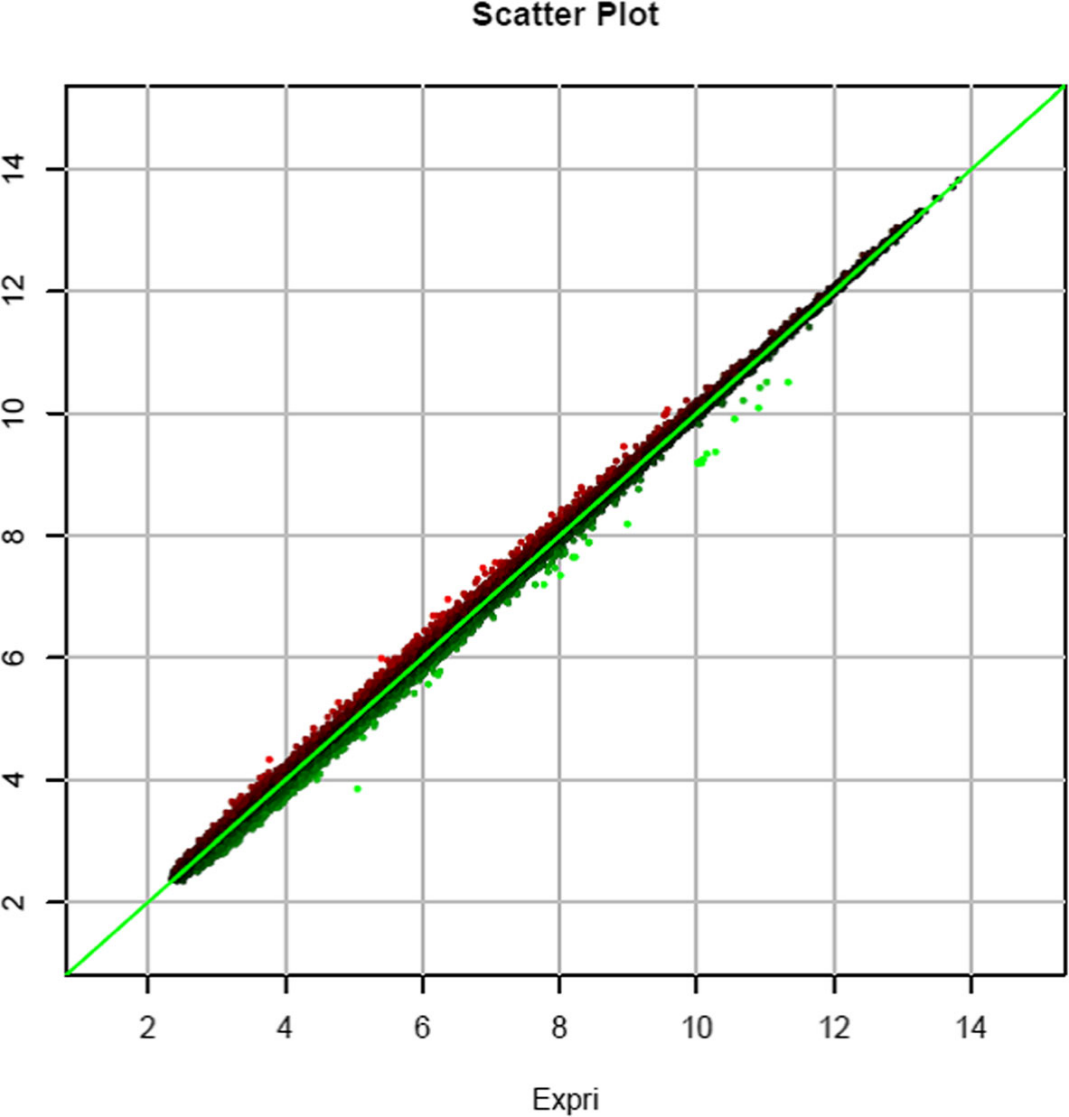
A principal component analysis scatter plot of differential gene expression patterns in ginsenoside-treated cells and control cells. Genes with equal expression values line up on the diagonal identity line and higher expression values are further away from the origin. Points below the diagonal represent genes with higher expression in ginsenoside-treated cells plotted on the *x*-axis. Points above the diagonal represent genes with higher expression values in control cells plotted on the *y*-axis. The further away a point-of-interest is from the diagonal line the larger is the difference in expression in ginsenoside-treated cells compared with the control cells

**Fig. 4:**
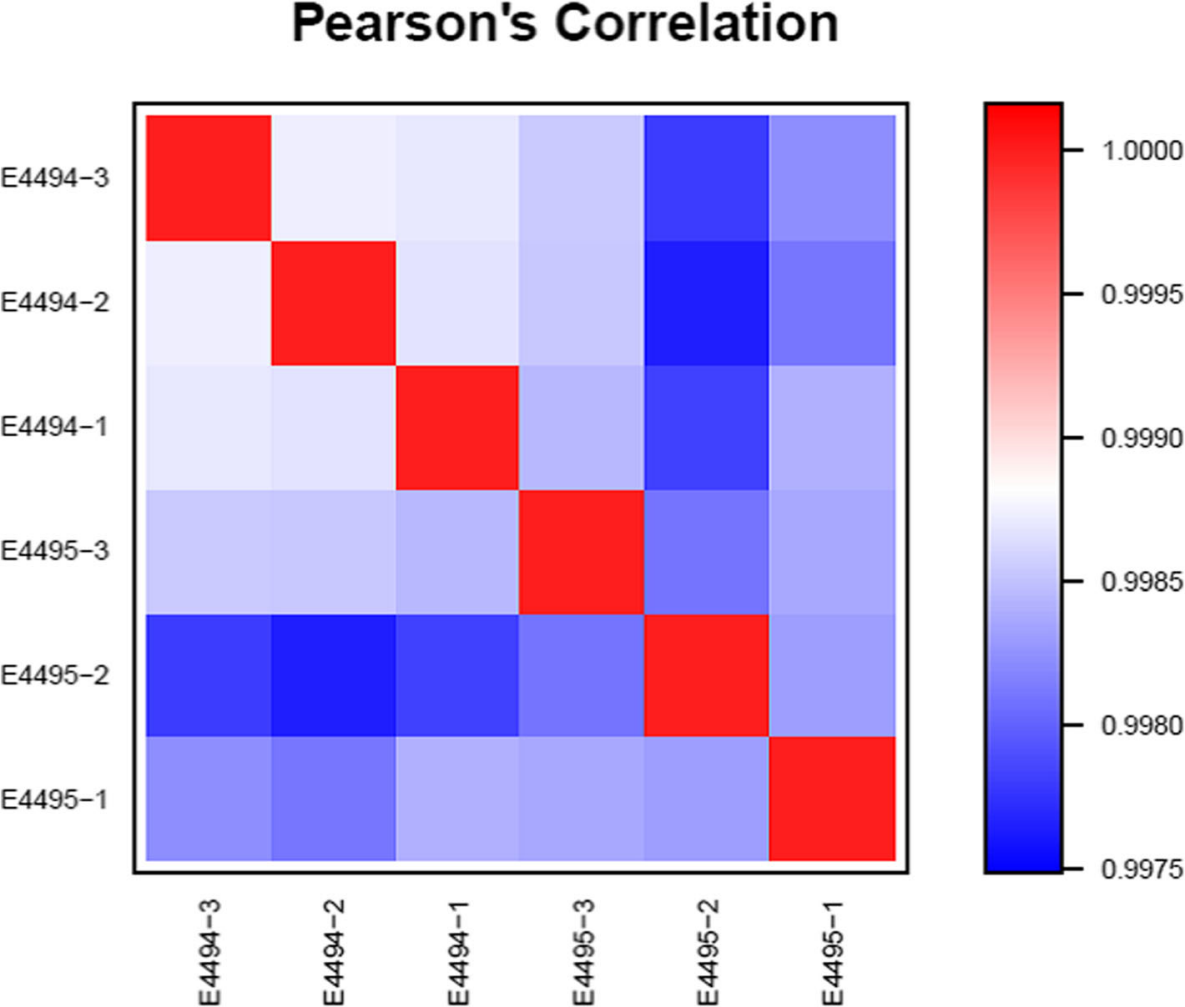
Correlation matrix of gene expression patterns in HLE-B3 cells treated with ginsenoside. The Pearson correlation was calculated between the vectors pointing from the overall mean of the entire dataset to the respective group mean. The values at each position in the matrix characterize the expression level of a particular gene under a particular experimental condition. Each box is a log ratio of gene expression of HLE-B3 cells treated with ginsenoside/the control cells. Rows represent genes and columns represent measurements from individual arrays

**Fig. 5:**
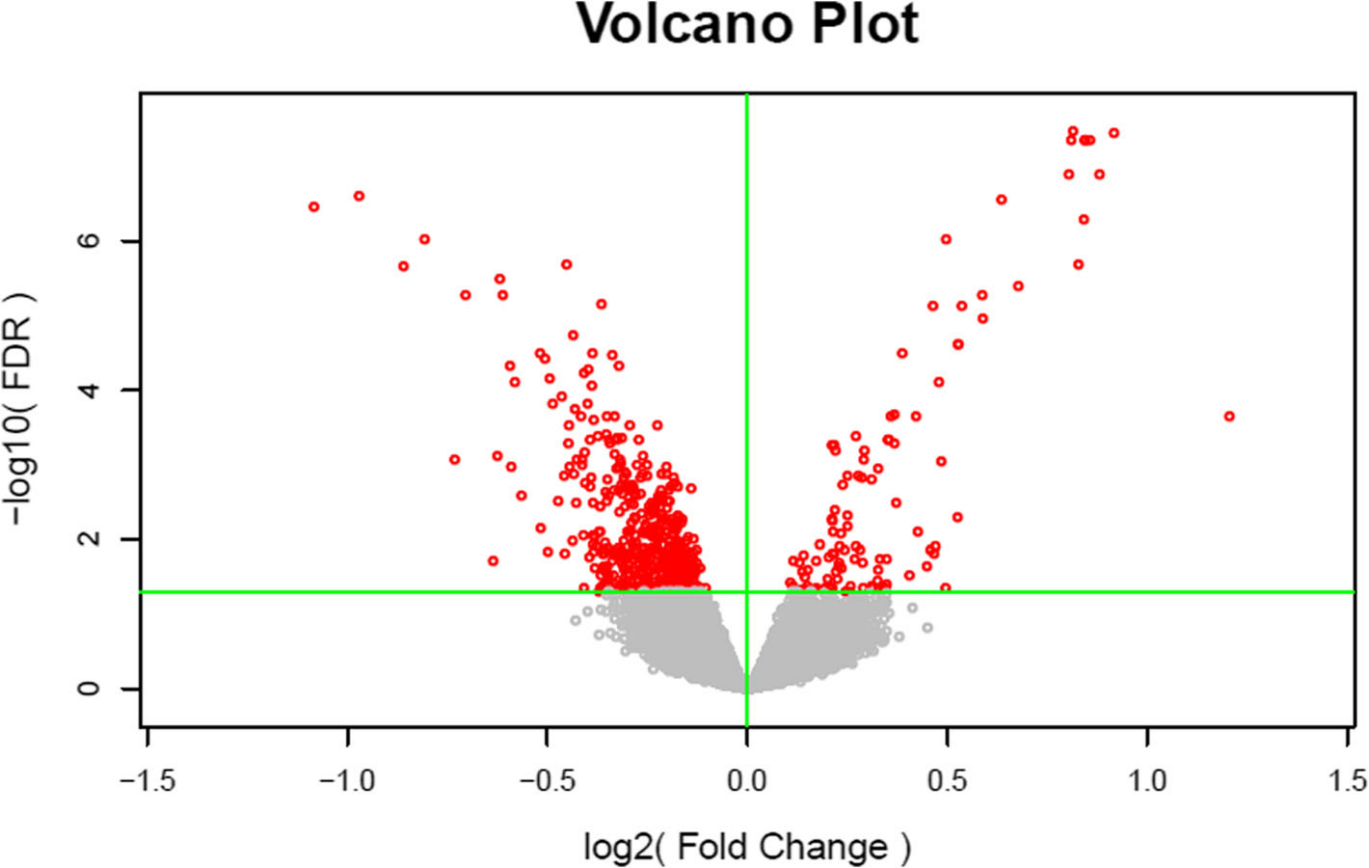
The volcano plot shows ginsenoside-induced changes of gene expression in HLE-B3 cells. The x axis shows fold changes in gene expression while the y axis shows statistical significance (−log10 of *P* values). The horizontal green line shows where *P* = 0.05 with points above the line having *P* < 0.05 and points below the line having *P* > 0.05. The volcano plot is color coded; those points having a fold-change less than 2 (log2 = 1) are shown in gray. Red indicates points-of-interest that display statistically significant differences. Points-of-interest left to the vertical green line represent downregulated genes and those that lie right to the vertical green line are upregulated genes

**Fig. 6:**
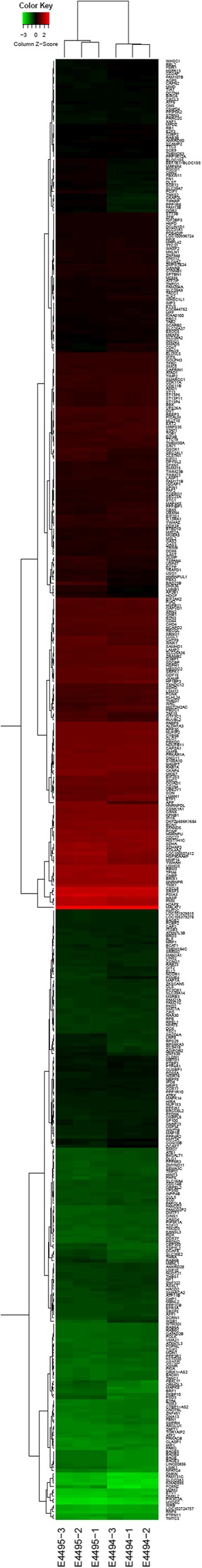
Hierarchical clustering. Genes in the data set were subjected to complete-linkage hierarchical clustering using a Euclidean distance metric. Genes that are upregulated appear in red, and those that are downregulated appear in green, with the relative log2 (ratio) reflected by the intensity of the color

Principal component analysis revealed that ginsenosides caused extensive changes in gene expression in HLE-B3 cells (Fig. 2). The differential gene expression patterns in ginsenoside-treated and control cells were further shown in a principal component analysis scatter plot (Fig. 3), indicating a linear relationship in gene expression patterns. The Pearson correlation matrix of gene expression patterns in HLE-B3 cells treated with ginsenosides is shown in Fig. 4. In addition, the volcano plot showed that more genes were downregulated than upregulated in ginsenosides-treated HLE-B3 cells (Fig. 5). Hierarchical clustering of gene expression patterns in HLE-B3 cells treated with ginsenosides further detailed the upregulated and downregulated genes (Fig. 6).

A total of 6219 genes showed significant differential expression in ginsenosides-treated and control HLE-B3 cells. Among them, 2552 (41.0%) genes were significantly upregulated while 3667 (59.0%) genes were significantly downregulated.

The 10 most upregulated and downregulated genes are shown in Tables 1 and 2, respectively. *FOXN2*, *APP* and *RAD23B* are the top three upregulated genes while *WSB1*, *PSME4* and *DCAF7* are the top three downregulated genes in HLE-B3 cells treated with ginsenosides. Ginsenoside caused one to two-fold increase in the ten most upregulated genes and one to two-fold decrease in the ten most downregulated genes in HLE-B3 cells.

**Table 1:** Ten most upregulated genes in HLE-B3 cells treated by ginsenoside

Gene Symbol	Gene Title	Fold Change	FDR	*P*-value
*FOXN2*	Forkhead box N2	2.304973238	0.000240794	2.54522E-07
*APP*	Amyloid beta (A4) precursor protein	1.887229663	2.83608E-08	1.14833E-12
*RAD23B*	RAD23 homolog B, nucleotide excision repair protein	1.745955352	1.08308E-07	1.66941E-11
*LMNB1*	Lamin B1	1.502640612	4.71803E-06	2.09592E-09
*MAPK8*	Mitogen-activated protein kinase 8	1.439462295	0.005637372	1.97442E-05
*LOC100507412*	Uncharacterized LOC100507412	1.411419323	8.33634E-07	2.36276E-10
*FBXL17*	F-box and leucine-rich repeat protein 17	1.410011243	0.050845053	0.000516737
*TLK2*	Tousled like kinase 2	1.394018783	1.394018783	0.47925
*PSD3*	Pleckstrin and Sec7 domain containing 3	1.382596329	0.017082448	9.57782E-05
*LIMK1*	LIM domain kinase 1	1.373614017	0.015861066	8.63777E-05

**Table 2:** Ten most downregulated genes in HLE-B3 cells treated by ginsenoside

Gene Symbol	Gene Title	Fold Change	FDR	P-value
*WSB1*	WD repeat and SOCS box containing 1	−2.1185	2.6E-07	5.9E-11
*PSME4*	Proteasome activator subunit 4	−1.814195587	1.8938E-06	6.13438E-10
*DCAF7*	DDB1 and CUL4 associated factor 7	−1.750123421	8.33634E-07	2.31236E-10
*ETV1*	Ets variant 1	−1.66127779	0.001099687	1.94511E-06
*SMAD5*	SMAD family member 5	−1.629545912	4.71803E-06	2.01829E-09
*DMXL2*	Dmx-like 2	−1.54160519	0.001079616	1.87968E-06
*CD47*	CD47 molecule	−1.535075911	3.03653E-06	1.10654E-09
*GIT2*	G protein-coupled receptor kinase interacting ArfGAP 2	−1.527296325	4.71803E-06	2.10136E-09
*SCRN1*	Secernin 1	−1.509209758	4.0976E-05	2.90345E-08
*NCOR1*	Nuclear receptor corepressor 1	−1.4958	7.4E-05	6E-08

### Ginsenosides upregulate the expression of genes involved in apoptosis and DNA damage response

Gene ontology analysis showed that three of the top ten upregulated genes were related to apoptosis, including *APP*, *LMNB1* and *MAPK8* (Table 3). Besides, three genes were involved in DNA damage response, including *RAD23B*, *MAPK8* and *TLK2*. Specifically, MAPK8 is involved in the cellular response to hydrogen peroxide and APP to OS. Furthermore, *PSME4*, which is one of the top ten downregulated genes, is involved in the negative regulation of apoptosis (Table 4).

**Table 3:** Annotated functions of ten most upregulated genes by ginsenoside

Gene Symbol	Biological Process	Cellular Component	Molecular Function
*FOXN2*	Transcription, regulation of transcription, skeletal muscle cell differentiation	Nucleus, nucleoplasm, intracellular membrane-bounded organelle	DNA binding, transcription factor activity, sequence-specific DNA binding
*APP*	Response to yeast, suckling behavior, platelet degranulation, mRNA polyadenylation, regulation of translation, protein phosphorylation, proteolysis, cellular copper ion homeostasis, post-Golgi vesicle-mediated transport, endocytosis, apoptotic process, response to oxidative stress, cell adhesion, regulation of epidermal growth factor-activated receptor activity, Notch signaling pathway, nervous system development, axonogenesis, blood coagulation, mating behavior, locomotory behavior, axon cargo transport, cholesterol metabolic process, adult locomotory behavior, visual learning, negative regulation of peptidase activity, regulation of gene expression, negative regulation of endopeptidase activity, positive regulation of G2/M transition of mitotic cell cycle, axon midline choice point recognition, neuron remodeling, dendrite development, antibacterial humoral response, antifungal humoral response, platelet activation, extracellular matrix organization, forebrain development, neuron projection development, ionotropic glutamate receptor signaling pathway, nucleotide-binding domain, leucine rich repeat containing receptor signaling pathway, regulation of multicellular organism growth, regulation of protein binding, cellular protein metabolic process, innate immune response, negative regulation of neuron differentiation, positive regulation of mitotic cell cycle, positive regulation of transcription from RNA polymerase II promoter, collateral sprouting in absence of injury, regulation of synapse structure or activity, defense response to Gram-negative and Gram-positive bacterium, neuromuscular process controlling balance, synaptic growth at neuromuscular junction, neuron apoptotic process, smooth endoplasmic reticulum calcium ion homeostasis, membrane organization	Extracellular region, extracellular space, nuclear envelope lumen, cytoplasm, endosome, smooth endoplasmic reticulum, Golgi apparatus, cytosol, plasma membrane, coated pit, cell-cell junction, cell surface, membrane, ER to Golgi transport vesicle, axon, platelet alpha granule lumen, cytoplasmic vesicle, neuromuscular junction, endosome lumen, trans-Golgi network membrane, ciliary rootlet, neuron projection, terminal bouton, dendritic spine, dendritic shaft, intracellular membrane-bounded organelle, receptor complex, membrane raft, apical part of cell, synapse, spindle midzone, extracellular exosome	DNA binding, serine-type endopeptidase inhibitor activity, receptor binding, protein binding, heparin binding, peptidase activity, peptidase activator activity, enzyme binding, peptidase inhibitor activity, acetylcholine receptor binding, metal ion binding, PTB domain binding, growth factor receptor binding,
*RAD23B*	Nucleotide-excision repair, DNA damage recognition, spermatogenesis, regulation of proteasomal ubiquitin-dependent protein catabolic process	Proteasome complex, nucleus, nucleoplasm, cytoplasm, XPC complex	Damaged DNA binding, single-stranded DNA binding, protein binding, polyubiquitin binding
*LMNB1*	Apoptotic process, cellular component disassembly involved in execution phase of apoptosis, programmed cell death	Nucleus, nuclear envelope, nuclear inner membrane, lamin filament, nucleoplasm, intermediate filament, membrane, nuclear matrix, nuclear membrane	Structural molecule activity, phospholipase binding
*MAPK8*	MAPK cascade, ossification, neuron migration, toll-like receptor signaling pathway, MyD88-dependent toll-like receptor signaling pathway, DNA repair, protein phosphorylation, apoptotic process, response to stress, JNK cascade, JUN phosphorylation, response to UV, regulation of gene expression, positive regulation of gene expression, programmed cell death, phosphorylation, peptidyl-serine phosphorylation, peptidyl-threonine phosphorylation, regulation of histone deacetylation, positive regulation of cyclase activity, negative regulation of protein binding, regulation of protein localization, toll-like receptor 2, 3, 4, 5, 9, and 10 signaling pathway, TRIF-dependent toll-like receptor signaling pathway, Fc-epsilon receptor signaling pathway, toll-like receptor TLR1:TLR2 and TLR6:TLR2 signaling pathway, regulation of circadian rhythm, positive and negative regulation of apoptotic process, innate immune response, response to cadmium ion, neurotrophin TRK receptor signaling pathway, rhythmic process, dendrite morphogenesis, regulation of sequence-specific DNA binding transcription factor activity, positive regulation of protein metabolic process, stress-activated MAPK cascade, cellular response to hydrogen peroxide, lipopolysaccharide, mechanical stimulus, and nitric oxide, positive regulation of deacetylase activity, apoptotic signaling pathway, intrinsic apoptotic signaling pathway, programmed necrotic cell death, positive regulation of protein insertion into mitochondrial membrane involved in apoptotic signaling pathway, positive regulation of determination of dorsal identity	Intracellular, nucleus, nucleoplasm, cytoplasm, mitochondrion, cytosol	Nucleotide binding, protein kinase activity, protein serine/threonine kinase activity, JUN kinase activity, MAP kinase activity, protein binding, ATP binding, kinase activity, transferase activity, transferring phosphorus-containing groups, enzyme binding, histone deacetylase regulator activity, histone deacetylase binding
*LOC1005*07412	–	–	–
*FBXL17*	Protein ubiquitination, SCF-dependent proteasomal ubiquitin-dependent protein catabolic process	Cytoplasm, SCF ubiquitin ligase complex	Protein binding, ubiquitin protein ligase activity
*TLK2*	Regulation of chromatin assembly or disassembly, protein phosphorylation, cellular response to DNA damage stimulus, cell cycle, chromosome segregation, negative regulation of autophagy, phosphorylation, chromatin modification, peptidyl-serine phosphorylation, negative regulation of proteasomal ubiquitin-dependent protein catabolic process, intracellular signal transduction, cellular response to gamma radiation	Nucleus, cytoplasm, cytoskeleton, intermediate filament, cell junction, perinuclear region of cytoplasm	Nucleotide binding, protein kinase activity, protein serine/threonine kinase activity, protein binding, ATP binding, kinase activity, transferase activity, transferring phosphorus-containing groups
*PSD3*	Vesicle-mediated transport, neuron differentiation, regulation of ARF protein signal transduction, positive regulation of GTPase activity	Trans-Golgi network, plasma membrane, postsynaptic density, membrane, cell junction, synapse, postsynaptic membrane	Guanyl-nucleotide exchange factor activity, ARF guanyl-nucleotide exchange factor activity, phospholipid binding
*LIMK1*	Protein phosphorylation, signal transduction, small GTPase mediated signal transduction, Rho protein signal transduction, nervous system development, axon guidance, actin cytoskeleton organization, positive regulation of actin filament bundle assembly, Fc-gamma receptor signaling pathway involved in phagocytosis, innate immune response, positive regulation of axon extension, ephrin receptor signaling pathway, negative regulation of ubiquitin-protein transferase activity, positive regulation of stress fiber assembly	Nucleus, nucleoplasm, cytoplasm, cytosol, focal adhesion, membrane, neuron projection	Nucleotide binding, protein kinase activity, protein serine/threonine kinase activity, protein binding, ATP binding, zinc ion binding, transferase activity, transferring phosphorus-containing groups, heat shock protein binding, metal ion binding, protein heterodimerization activity

**Table 4:** Annotated functions of ten most downregulated genes by ginsenoside

Gene Symbol	Biological Process	Cellular Component	Molecular Function
*WSB1*	Phosphorylation, protein ubiquitination, intracellular signal transduction	Intracellular	Protein binding, kinase activity
*PSME4*	MAPK cascade, activation of MAPKK activity, protein polyubiquitination, mitotic cell cycle, stimulatory C-type lectin receptor signaling pathway, antigen processing and presentation of peptide antigen via MHC class I, antigen processing and presentation of exogenous peptide antigen via MHC class I, TAP-dependent, DNA repair, regulation of cellular amino acid metabolic process, polyamine metabolic process, apoptotic process, cellular response to DNA damage stimulus, DNA damage response, signal transduction by p53 class mediator resulting in cell cycle arrest, epidermal growth factor receptor signaling pathway, small GTPase mediated signal transduction, Ras protein signal transduction, multicellular organismal development, spermatogenesis, axon guidance, insulin receptor signaling pathway, fibroblast growth factor receptor signaling pathway, gene expression, proteasomal ubiquitin-independent protein catabolic process, positive regulation of peptidase activity, programmed cell death, viral process, cell differentiation, anaphase-promoting complex-dependent proteasomal ubiquitin-dependent protein catabolic process, tumor necrosis factor-mediated signaling pathway, cellular nitrogen compound metabolic process, spermatogenesis, exchange of chromosomal proteins, NIK/NF-kappaB signaling, Fc-epsilon receptor signaling pathway, antigen processing and presentation of exogenous peptide antigen via MHC class I, regulation of apoptotic process, negative regulation of apoptotic process, regulation of mRNA stability, small molecule metabolic process, innate immune response, vascular endothelial growth factor receptor signaling pathway, neurotrophin TRK receptor signaling pathway, T cell receptor signaling pathway, positive regulation of ubiquitin-protein ligase activity involved in regulation of mitotic cell cycle transition, regulation of ubiquitin-protein ligase activity involved in mitotic cell cycle, negative and positive regulation of canonical Wnt signaling pathway	Proteasome complex, nucleus, cytoplasm, cytosol, spermatoproteasome complex	Protein binding, peptidase activator activity, lysine-acetylated histone binding
*DCAF7*	Multicellular organismal development, protein ubiquitination, protein ubiquitination	Nucleus, cytoplasm, cytoplasm, nuclear matrix, protein complex, Cul4-RING E3 ubiquitin ligase complex	Protein binding
*ETV1*	Transcription, regulation of transcription, transcription from RNA polymerase, axon guidance, muscle organ development, mechanosensory behavior, cell differentiation, positive regulation of transcription, positive regulation of transcription from RNA polymerase II promoter, peripheral nervous system neuron development	Nucleus	RNA polymerase II core promoter proximal region sequence-specific DNA binding, transcriptional activator activity, RNA polymerase II core promoter proximal region sequence-specific binding, DNA binding, transcription factor activity, sequence-specific DNA binding, protein binding, sequence-specific DNA binding
*SMAD5*	Ossification, ureteric bud development, Mullerian duct regression, osteoblast fate commitment, transcription, regulation of transcription, protein phosphorylation, signal transduction, transforming growth factor beta receptor signaling pathway, germ cell development, embryonic pattern specification, erythrocyte differentiation, BMP signaling pathway, intracellular signal transduction, positive regulation of osteoblast differentiation, positive regulation of transcription, positive regulation of transcription from RNA polymerase II promoter, cartilage development, cardiac muscle contraction, bone development, SMAD protein signal transduction, cellular response to organic cyclic compound, cellular response to BMP stimulus, positive regulation of transcription from RNA polymerase II promoter involved in cellular response to chemical stimulus	Intracellular, nucleus, nucleoplasm, nucleoplasm, transcription factor complex, cytoplasm, cytosol, integral component of membrane, protein complex, SMAD protein complex	RNA polymerase II core promoter sequence-specific DNA binding, DNA binding, transcription factor activity, sequence-specific DNA binding, receptor signaling protein activity, protein binding, transforming growth factor beta receptor, pathway-specific cytoplasmic mediator activity, ubiquitin protein ligase binding, metal ion binding
*DMXL2*	–	Extracellular space, synaptic vesicle, membrane, cell junction, synaptic vesicle membrane, cytoplasmic vesicle, synapse	Protein binding, Rab GTPase binding
*CD47*	Cell adhesion, integrin-mediated signaling pathway, blood coagulation, opsonization, positive regulation of cell proliferation, response to bacterium, positive regulation of cell-cell adhesion, extracellular matrix organization, positive regulation of inflammatory response, positive regulation of phagocytosis, positive regulation of T cell activation, leukocyte migration	Plasma membrane, integral component of plasma membrane, membrane, integral component of membrane, extracellular exosome	protein binding, thrombospondin receptor activity
*GIT2*	Regulation of G-protein coupled receptor protein signaling pathway, phosphorylation, positive regulation of GTPase activity, behavioral response to pain	Nucleoplasm, focal adhesion	GTPase activator activity, protein binding, kinase activity, protein complex binding, metal ion binding
*SCRN1*	Proteolysis, exocytosis	Cell, nucleus, cytoplasm, nuclear membrane	Protein binding, dipeptidase activity
*NCOR1*	Negative regulation of transcription from RNA polymerase II promoter, regulation of thyroid hormone mediated signaling pathway, CD4-positive, CD25-positive, alpha-beta regulatory T cell differentiation, chromatin organization, regulation of transcription, transcription initiation from RNA polymerase II promoter, transforming growth factor beta receptor signaling pathway, Notch signaling pathway, circadian rhythm, gene expression, negative regulation of phosphatidylinositol 3-kinase signaling, chromatin modification, thalamus development, positive regulation of histone deacetylation, circadian regulation of gene expression, regulation of multicellular organism growth, cholesterol homeostasis, cellular lipid metabolic process, small molecule metabolic process, negative regulation of JNK cascade, spindle assembly, definitive erythrocyte differentiation, regulation of glycolytic process, regulation of lipid transport, cellular response to thyroglobulin triiodothyronine, regulation of fatty acid transport	Histone deacetylase complex, nuclear chromatin, nucleus, nucleoplasm, transcription factor complex, cytoplasm, spindle microtubule, membrane, Sin3 complex, transcriptional repressor complex	RNA polymerase II activating transcription factor binding, DNA binding, chromatin binding, transcription factor activity, transcription corepressor activity, protein binding, ligand-dependent nuclear receptor binding, histone deacetylase regulator activity, nuclear hormone receptor binding, histone deacetylase binding, retinoic acid receptor binding, sequence-specific DNA binding, transcription regulatory region DNA binding, retinoid X receptor binding, thyroid hormone receptor binding

### Ginsenosides reduce H_2_O_2_-induced apoptosis of HLE-B3 cells

**Fig. 7:**
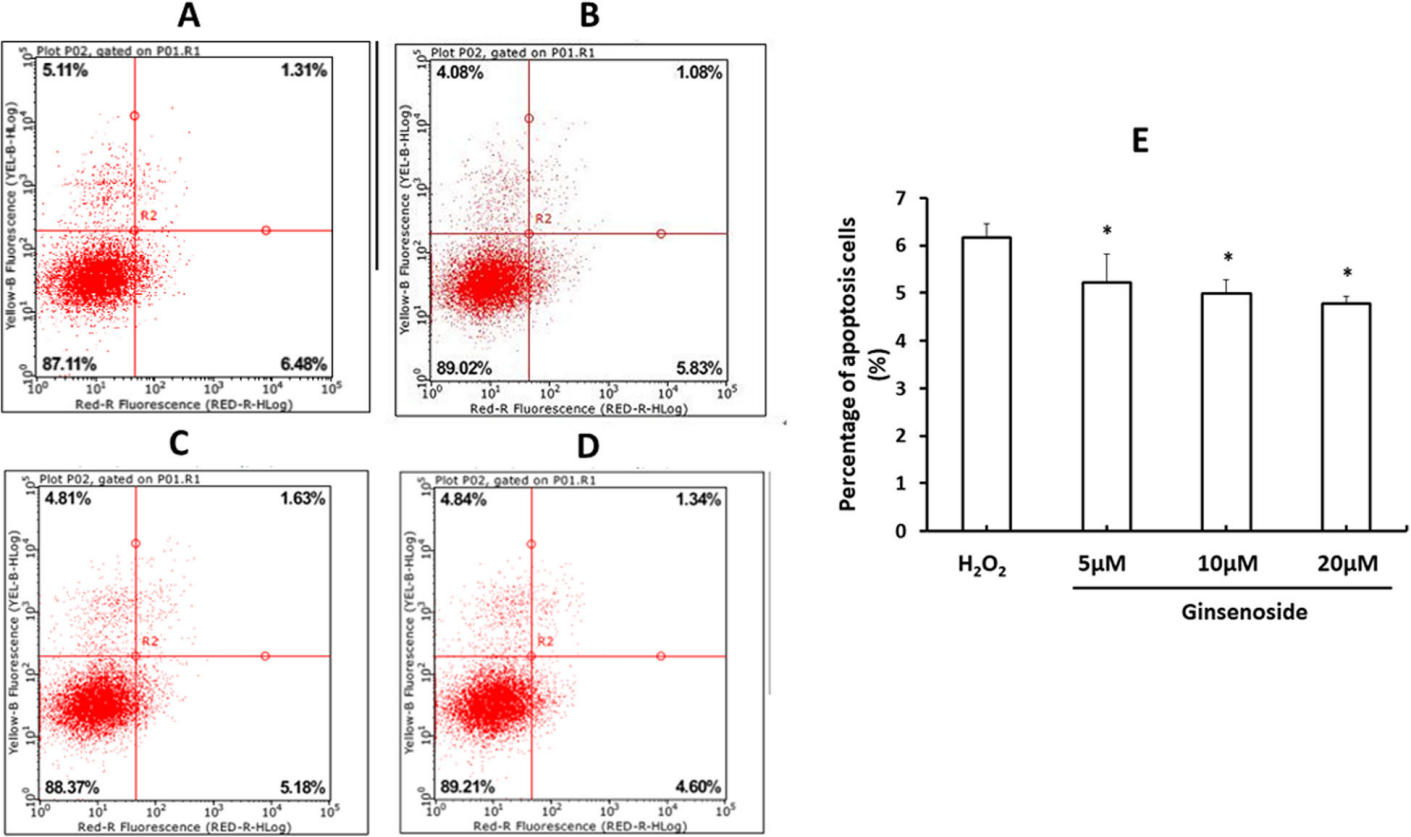
Ginsenoside reduces H_2_O_2_-induced apoptosis of HLE-B3 cells. HLE-B3 cells were treated with 1.75 μM H_2_O_2_ (**a**) and low (5 μM) (**b**), mid (10 μM) (**c**) and high dose (20 μM) (**d**) of ginsenoside. The percentage of apoptosis cells was examined by flow cytometry. **e** Data are expressed as mean ± SD of at least three independent experiments. *P* < 0.05 of the treatment groups versus 1.75 μM H_2_O_2_. Representative histograms are shown in (**a**) to (**d**)

Since gene ontology analysis revealed that ginsenosides modulated the expression of apoptosis-related genes, such as *LMNB1* and *PSME4*, we examined the effect of ginsenosides on H_2_O_2_-induced apoptosis of HLE-B3 cells*.* Flow cytometry analysis showed that 6.16 ± 0.29% of H_2_O_2_-treated HLE-B3 cells were early apoptotic cells (Fig. 7a), and the percentage was significantly reduced to 5.22 ± 0.59%, 4.98 ± 0.29% and 4.78 ± 0.16% by the presence of low (5 μM), mid (10 μM) and high (20 μM) dose of ginsenosides (*P* < 0.05), respectively (Fig. 7b to e), suggesting that ginsenosides reduce H_2_O_2_-induced apoptosis in HLE-B3 cells.

## Discussion

In this study, we presented the first experimental evidence that ginsenosides could protect against H_2_O_2_-induced growth inhibition and apoptosis in HLE-B3 cells. Furthermore, ginsenosides caused widespread changes in gene expression, including changes in genes involved in DNA damage response and apoptosis, suggesting that ginsenosides act through multiple molecular mechanisms.

Microarray data showed that *FOXN2*, *APP* and *RAD23B* were the top three upregulated genes while *WSB1*, *PSME4* and *DCAF7* were the top three downregulated genes by ginsenosides in HLE-B3 cells. FOXN2 is a member of the Forkhead box transcription factors. Its role in cataractogenesis has not been confirmed. A recent study showed that FOXN2 could suppress the proliferation of lung cancer cells [17]. *RAD23B*, *MAPK8* and *TLK2* have been shown to be involved in DNA damage response [18]. Effect of ginsenosides on MAPK has been well documented [19–21]. A Chinese herbal medicine containing ginsenosides was found to attenuate H_2_O_2_-induced injury in PC12 cells by inhibiting Akt and MAPK signaling pathways [22]. H_2_O_2_ in the aqueous humor can activate MAPK signaling in HLE cells [7, 23], but the exact effect of ginsenosides on MAPK in HLB cells remains to be elucidated.

We also showed that ginsenosides reduced the percentage of early apoptotic HLB cells. This is consistent with our finding that ginsenosides modulate the expression of apoptosis-related genes such as *LMNB1* and *PSME4.* In fact, *PSME4* is among the top ten downregulated genes and is involved in negative regulation of apoptosis. In addition, three of the top ten upregulated genes are also related to apoptosis, including *APP*, *LMNB1* and *MAPK8*. Apoptosis of HLB cells is increased during cataractogenesis [24], while ginsenosides may attenuate cataractogenesis by inhibiting H_2_O_2_–induced expression of apoptosis-related genes in HLB cells.

## Conclusion

Ginsenosides can induce widespread changes in the expression of genes involved in multiple signaling pathways, including apoptotic signaling and DNA damage response signaling. Ginsenosides can alleviate H_2_O_2_-induced growth inhibition and inhibit H_2_O_2_-induced apoptosis in HLB cells.

## Data Availability

The datasets supporting the conclusions of this article are included within the article.

## Abbreviations

H_2_O_2_: Hydrogen peroxide
HLE: Human lens epithelial
MAPKs: Mitogen-activated protein kinases
OS: Oxidative stress
PBS: Phosphate-buffered saline
ROS: Reactive oxygen species
